# Age-specific olfactory attraction between Western honey bee drones (*Apis mellifera*) and its chemical basis

**DOI:** 10.1371/journal.pone.0185949

**Published:** 2017-10-04

**Authors:** Florian Bastin, Fabrice Savarit, Grégory Lafon, Jean-Christophe Sandoz

**Affiliations:** 1 Evolution, Genomes, Behaviour and Ecology, CNRS (UMR 9191), Univ Paris-Sud, IRD, Université Paris-Saclay, Gif-sur-Yvette, France; 2 Laboratoire d’Ethologie Expérimentale et Comparée, Université Paris 13, Sorbonne Paris Cité, Villetaneuse, France; University of Arizona, UNITED STATES

## Abstract

During the mating season, drones (males) of the Western honey bee (*Apis mellifera*) form congregations numbering thousands high in the air. Virgin queens arrive at these congregations after they have formed and mate on the fly with 15-20 drones. To explain the formation of drone congregations, a drone-produced aggregation pheromone has been proposed many years ago but due to the low accessibility of natural mating sites in bees, its study has progressed slowly. Recently, we used a walking simulator in controlled laboratory conditions to show that drones are indeed attracted by groups of other drones. Since these previous experiments were carried out with drones captured when flying out of the hive, it is currently unclear if this olfactory attraction behaviour is related to the drones’ sexual maturity (usually reached between 9 and 12 days) and may thus be indicative of a possible role in congregation formation, or if it is observed at any age and may represent in-hive aggregation. We thus assessed here the dependency of drone olfactory attraction on their age. First, we performed behavioural experiments in the walking simulator to measure olfactory preferences of drones in three age groups from 2-3 to 12-15 days. Then, we performed chemical analyses in the same age groups to evaluate whether chemical substances produced by the drones may explain age differences in olfactory attraction. We show that honey bee drones are attracted by conspecifics of the same age when they are sexually mature (12-15 days old) but not when they are younger (2-3 and 7-8 days old). In parallel, our data show that drones’ chemical profile changes with age, including its most volatile fraction. These results are discussed in the context of drone mutual attraction both within the hive and at drone congregations.

## Introduction

Bee pollination contributes to agriculture productivity and biodiversity. The Western honey bee *Apis mellifera* is the most economically important pollinator in the world [1–2]. For many years now, honey bees have also become a main-stream animal model for scientific studies in genetics, physiology, ethology, neurobiology and animal cognition [3–11]. Honey bee reproduction has been studied for decades and its mating behaviour has fascinated many scientists [12–18]. On warm and sunny afternoons during the mating season, sexually mature honey bee males, the drones, fly out of the nest and gather high in the air at discrete congregation areas which can contain as many as 11000 drones from up to 240 different colonies [19–21]. Drone congregations are between 30 and 200 m in diameter, located 10–40 m above ground [22–25]. About one hour after the peak of drones’ departure from the hives, virgin queens also leave their hive and join the drone congregation [13,25,26]. As soon as a virgin queen enters the congregation, groups of drones are attracted to her, first by olfactory cues (pheromones) and at shorter range by visual cues [27]. Drones follow the virgin queen in a comet-like swarm and engage in a scramble competition, each individual struggling for the most promising position to approach and mate with the queen [27]. Usually, a queen mates within 15–30 minute with 10–20 drones, who die directly after mating [20,28,29].

It is widely acknowledged that honey bee reproduction is mediated by pheromones [18]. Pheromones are volatile chemicals used for communication between individuals of the same species [30]. Honey bees, like many insects, employ a rich repertoire of pheromones to ensure intraspecific communication in many behavioural contexts [19,31–33]. In the context of mating, the major compound of the queen mandibular pheromone (QMP), 9-oxo-2-decenoic acid (9-ODA), which also participates in communicating the queens’ fertility within the hive [31,34,35], attracts drones over long distances [36–40]. It possibly acts together with secondary components also involved in the communication between queens and workers [41]. While the existence of this pheromone explains how drones find the virgin queens for mating, it does not explain the formation of the drone congregations. The presence of a queen is not necessary for forming a drone congregation since, as indicated above, queens join the congregation after the drones [13,25,26]. Although local (visual) cues are probably involved, a drone-produced aggregation pheromone has been proposed to explain the formation of drone congregations [19,42,43]. However, because of obvious limitations due to the low accessibility of drone congregations located high in the air, the exact cues used by the drones to find the congregations have remained unclear. To study the possible role of olfactory cues in controlled laboratory conditions, we recently developed a walking simulator assay allowing to explore drones’ olfactory orientation behaviour [44]. We could show that drones captured when flying out of the hive are indeed attracted to the odour bouquet from other drones [44]. Interestingly, virgin queens are also attracted to such drones’ odour bouquet in this setup [45]. These results confirmed that drones produce an attractive odour substance. However, because our previous experiments used drones captured when flying out of the colony, it is unclear if the observed olfactory attraction relates to these drones’ sexual maturity, which evolves with age, and may thus be indicative of a possible role in congregation formation, or if it also exists in younger drones and may correspond to in-hive aggregation behaviour.

Honey bee drones are produced by the queen only during the reproduction period (spring and summer). The only known role of the drones is reproduction, although they may occasionally take part in thermoregulation during extreme thermal stress conditions [46,47]. In the hive, they do not contribute to the workers’ tasks but spend 70% of their time immobile on the comb, and the rest of the time they consume food stored in the hive [48]. During their first days of life, drones are fed by young workers through trophallaxis on the central combs where temperature is the warmest [48–51]. When they become older, drones move toward peripheral combs and feed themselves directly on honey, gathering sufficient energy for nuptial flights [48]. Drones of all ages may be found aggregated in small groups [49,51]. Before winter or in case of scarce resources, drones are removed from the hive by workers [52,53]. Older drones are usually ejected before the young drones [48], suggesting that workers can discriminate between these age groups, most probably through olfactory cues. Among these, non-volatile cues (cuticular hydrocarbons) were indeed shown to evolve with drones’ age [54]. How more volatile cues change with age is as yet unknown.

Two main characteristics of drone sexual maturity, sperm quality and flight capacity, which are both indispensable for successful mating, have been examined. Many studies placed drones’ sexual maturity at their first flight out of the hive, i.e. between 7 and 9 days of age [55–58]. However, the first flights made by drones are hygienic (defecation) and orientation flights, and are much shorter (1–6 min) than mating flights (32 ± 22 min), during which drones visit congregation areas and attempt to mate with a queen [18,22,59]. According to these last studies, sexual maturity would rather be achieved at the onset of real mating flights, between 10 and 12 days of age. On a physiological level, sperm maturation takes place during the first days of adult life. Starting at three days of age, spermatozoa are transferred to the seminal vesicles and reach their highest number when the drones turn 8 to 12 days old, depending on the studies [18,60]. The secretions of endophallus accessory glands, including mucus and corneal glands, also mature, being fully functional at 9–12 days [18,61,62]. Taken together, data on sperm maturation and flight activity both suggest that drones are not fully sexually operational before 10–12 days of age. How drones’ age – and sexual maturity—affect their olfactory mutual attraction is currently unknown.

In the present study, we thus asked whether drones’ olfactory attraction toward other drones is age-specific. First, we performed behavioural experiments to measure olfactory preferences of drones in three age groups from 2–3 days to 12–15 days. Then, we performed chemical analyses in the same age groups to evaluate whether chemical substances produced by drones may explain age differences in olfactory attraction. We show that honey bee drones are attracted by conspecifics of the same age when they are sexually mature (12–15 days old) but not when they are younger (2–3 and 7–8 days old). In parallel, our data show that drones’ chemical profiles change with age, including its most volatile fraction. These results pave the way for the identification of semiochemicals involved in drone mutual attraction.

## Methods

### Insects

Walking simulator experiments were performed with drone honey bees (*Apis mellifera*) of controlled age. Drones were obtained from the CNRS campus in Gif-sur-Yvette (France). All experiments were performed from 10:00 to 20:00 during the reproductive season, between May and September. Pre-emerging drone brood combs were placed into an incubator (34°). Drones were marked at emergence with a specific colour code on the thorax, using water-based paint (Posca PC-5M, Mitsubishi Pencil Co.) and were then introduced back into their original hive. Depending on the requested age for each experiment, drones bearing the corresponding colour code were caught with forceps directly on the combs after opening the hive. Three age groups were studied: 2–3 days, 7–8 days and 12–15 days.

### Behavioural experiments

#### Bee fixing

Drones caught from the hive on the day of the experiment were placed in groups in plexiglas cages [63] containing a piece of wax comb and providing honey and water *ad libitum*. Before the experiment, a very small insect needle (minutens 3.20, Ento Sphinx, Pardubice, Czeck Republic) was glued to the thorax of a drone using low-temperature melting wax (Deiberit 502; Schöps and Dr. Böhme, Goslar, Germany) or UV-reactive glue (3M ESPE Sinfony opaque dentine 3, Cergy-Pontoise, France) and a curing light (Woodpecker LED.B, Guilin, Guangxi, PR China). The drone was then attached in the setup by means of this needle, so that it rested lightly on the walking simulator.

#### Walking simulator

**Fig 1:**
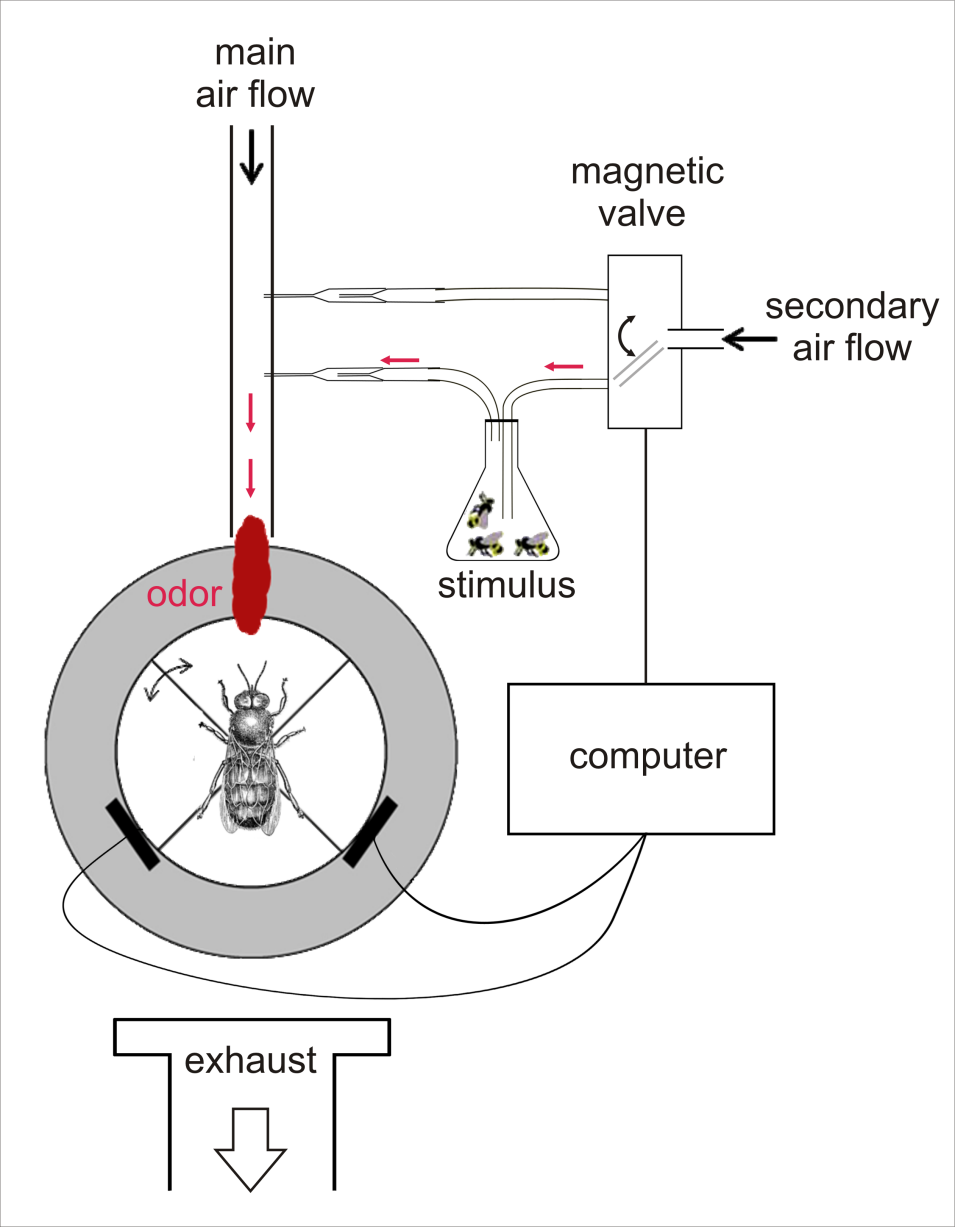
Walking simulator setup. A tethered honey bee drones is allowed to freely walk on an air-supported ball (in white). The drone can easily turn the ball under himself. Ball displacement is recorded via two computer-mouse sensors (black bars close to the ball), which allow reconstructing the drone’s walking path. Odour stimulation is provided via a main, constant, air-stream directed to the drone. Odours are quickly removed from the setup by an exhaust behind the drone. All experiments were conducted in complete darkness. The ball is virtually divided into 4 quadrants, one of which is designated as the odour quadrant. After an accommodation phase of 5 min (‘before’ phase), stimulus control is granted to the bee for 5 min (‘during’ phase): whenever the drone’s virtual heading is in the odour quadrant (as shown on the figure), odour stimulation is activated using the computer-controlled magnetic valves. This allows quantifying, whether the animal preferred receiving odour stimulation or not. A group of 10 drones of the same age as the focal drone (i.e. on the ball), were used as stimuli.

We used a walking simulator to test the odour preferences of age-controlled drones (Fig 1). The setup consists of an air-supported ping-pong ball (Cornilleau Competition, Breteuil, France; diameter: 40 mm; weight: 2.7g), on which a tethered insect can freely walk (but cannot fly) in any virtual direction by turning the ball below it. As a ball holder, we used a custom-made Plexiglas block with a hemispherical cavity slightly larger than the ping-pong ball. An air inlet at the bottom of the cavity allowed the ball to float on an air cushion. Because of the custom-made ball holder design, only a weak air stream was needed to support the ball sufficiently and, hence, no disturbing air currents were detectable in the vicinity of the drone. Air flow was precisely controlled using a pressure regulator (Air Liquide REC BS 50-1-2, Paris, France). The air was filtered using activated charcoal (Sigma-Aldrich Norit RB1, Steinheim, Germany). An air extractor was placed behind the bee to avoid any odour contaminations of the setup. All experiments were performed in complete darkness under an opaque cage protecting the setup from stray light and undesired air currents.

#### Recording

Two highly-sensitive optical sensors from laser mice were used to record ball movements (Logitech G500, Morges, Switzerland: resolution: 5700 dpi, signal rate: 1000 Hz). They were attached to the Plexiglas block at the horizontal equator of the ball and at a relative angle of 90° to each other. The body axis of the insect was always precisely aligned at an angle of 45° with respect to both mouse sensors. Mouse signals were integrated and recorded via custom-written software written in LabView 2011 (National Instruments, Nanterre, France) using ManyMouse to separately handle the signals of both mouse sensors (source code by Ryan C. Gordon; http://icculus.org/manymouse). From the recorded ball movements, custom-written software directly calculated the bee’s walking path, and produced throughout the experiment several parameters such as its walking speed, turning direction and virtual heading.

#### Odour stimulation

We applied the protocol from the second experiment in [44]. A continuous airflow, into which odour stimulations could be applied, was delivered directly in front of the bee. The air flow was delivered by a glass tube (inner diameter: 7 mm) at a distance of 20 mm in direction of the drone’s antennae (Fig 1). The air flow consisted of a main continuous air flow (1 L/h) and a secondary air flow (0.2 L/h), which were filtered by activated charcoal (Sigma-Aldrich Norit RB1) and regulated by flow-meters (Brooks Instrument Model 1355E Sho-rate, R-2-15-D and R-2-15-AAA respectively, Hatfield, PA, USA). An odour stimulation could be applied using computer controlled magnetic valves (Lee LFAA1200118H, Voisins Le Bretonneux, France; controlled via a BMCM R8 relay and USB-PIO, Maisach, Germany), switching the secondary continuous airflow from an empty Pasteur pipette to an odour pipette attached to a vial containing stimulation animals. Due to the fast switching magnetic valves between control pipette and odour pipette, total air flow in front of the bee was held at a constant rate of 1.2 L/h. As olfactory stimulation, we used the odour bouquet from 10 living drones placed in a 100 mL vial which was connected to the odour Pasteur pipette of the secondary air flow (Fig 1). The drones used for stimulation had the same age as the tested drones. Each drone was tested only once in the walking simulator. When the activity of the stimulus drones was decreasing in the stimulation vial (after about 3 h), they were replaced with new ones.

#### Experimental protocol

Each experiment consisted of two periods of 5 minutes each. First, bees received a 5 min accommodation to the experimental setup, during which they could freely walk on the ball ('before' phase). After that time, an odour pulse of 1 sec was delivered to signal to the bee the presence of an odour cue in the setup. Then the full control over odour stimulation was given to the bee during 5 minutes ('during' phase). To this end, the ball was virtually divided into 4 quadrants, and one quadrant was pseudorandomly designated as the odour quadrant. Odour stimulation was activated whenever the bee was heading toward the virtual odour quadrant (Fig 1). Thus, the tested drone received a clear feedback from its own behaviour (closed loop). Because turning the ball is an easy task for the bee, we can evaluate whether it preferred to receive odour stimulation or not. As shown by a previous experiment testing workers with an appetitively learned odorant (supplementary material in [45]), attraction can be measured by the time spent and the distance walked by the insect in the odour quadrant relative to the other quadrants.

### Chemical analysis

In total, 151 drones were subjected to chemical analysis (2–3 days old: 79; 7–8 days old: 28; 12–15 days old: 44). These drones were from the same origin as the experimental drones, but were never used in the behavioural experiments. To extract their body odour, age-controlled drones were placed in a freezer (-21°C) for at least 15 min. Then they were transferred individually into 4 mL glass tubes (Interchim, Montluçon, France). Each drone was immersed in 1 mL pentane (Sigma Aldrich) for 10 min with 15 s vortexing at the beginning. Then, 400 μL of the extract were transferred to 2 mL glass tubes with Teflon steal stoppers (Interchim, Montluçon, France) and stored at -21°C until analysis. After evaporation and concentration of extracts in inserts of 250 μL (Interchim, Montluçon, France), 2 μL were injected in a gas chromatograph coupled with a mass spectrometer. The samples were analyzed on an HP Agilent 7890A gas chromatograph equipped with an HP-5ms column (30 m x 250 μm x 0.25 μm), coupled to an HP Agilent 5975C inert XL mass spectrometer (with -70 eV electron impact ionization). The liquid samples were injected at 280°C in splitless mode for 1 min. Carrier gas was helium at a constant flow rate of 1 mL/min. After 5 min hold at 50°C, the oven was programmed to increase the column temperature from 50°C to 200°C at 6°C/min, then from 200°C to 320°C at 20°C/min and then to keep the temperature constant at 320°C for 5 min.

### Data analysis and statistics

#### Behavioural data

To evaluate the effect of drone maturation on their mutual olfactory attraction, the behavioural experiments were carried out with three groups of drones: 2–3 day-old drones (N = 59), 7–8 day-old drones (N = 46) and 12–15 day-old drones (N = 39). To ensure that the results reflected the behaviour of fit, well-positioned and closed-loop aware drones, three selection criteria were used [45]: 1) Mobility: unfit drones, i.e. individuals that walked less than 200 mm during the first 5 min, were excluded; 2) Lateral bias: drones that turned more than 7200° (i.e. 20 full turns) in any one direction during the first 5 min were also excluded, as they were either fixed in an inadequate position on the ball or were too strongly lateralized. 3) Closed loop: because the experiment aims to measure insects’ behavioural choice to receive or not the odorant stimulation, the drones that never experienced their own control over odour delivery cannot be kept [44]. Thus, individuals that never toggled the odour ON or OFF through their own behaviour during the stimulus control (i.e. 'during') phase, i.e. drones that never switched from an odour quadrant to a non-odour quadrant (and vice versa), were excluded. Overall, 29% of 2–3 day-old drones, 38% of 7–8 day-old drones, 11% of 12–15 day-old drones were thus excluded from the analysis.

**Fig 2:**
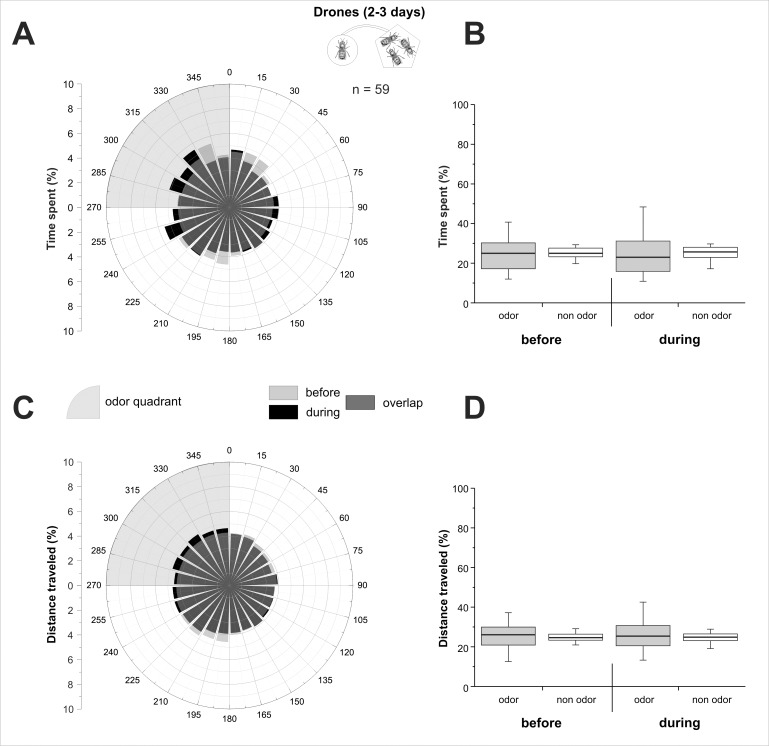
Olfactory attraction between 2–3 day-old drones. 2–3 day-old drones’ behaviour on the walking simulator, when stimulated with the odour bouquet of 10 same age drones. **A,C)** Circular histograms showing the percentage of time spent (A) or of distance travelled (C) by drones according to 15° sectors, with the odour quadrant being represented on the upper left (grey area). Light grey bars represent the 5 min before odour stimulation (‘before’), black bars represent the 5 min during stimulation (‘during’), and hence, dark grey bars show the overlap of the two phases. **B,D)** Histograms of the percentage of time spent (B), or of distance travelled (D) by drones in the odour quadrant (gray box) and on average in the three odourless quadrants (white box) before and during odour stimulation.

**Fig 3:**
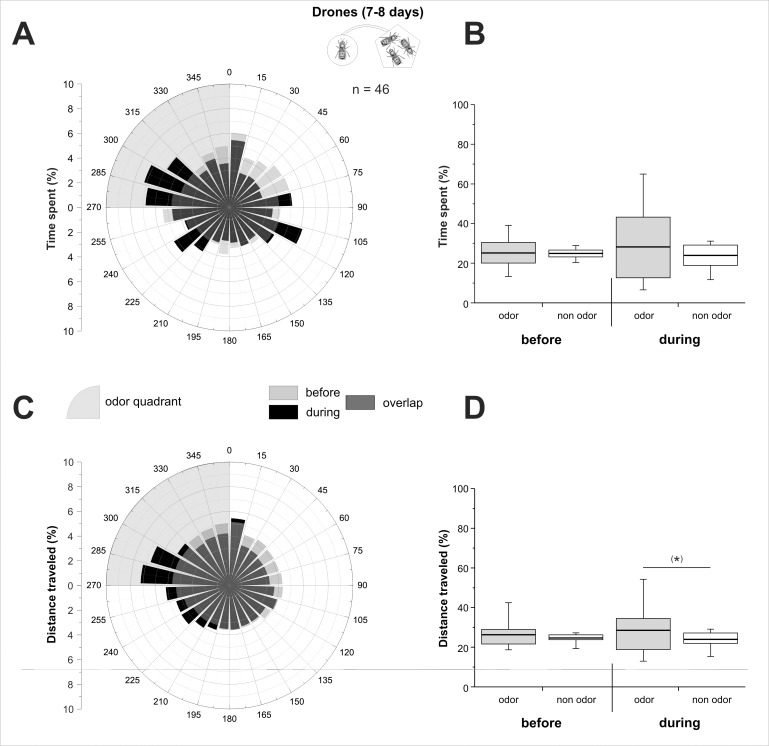
Olfactory attraction between 7–8 day-old drones. 7–8 day-old drones’ behaviour on the walking simulator, when stimulated with the odour bouquet of 10 same age drones. **A,C)** Circular histograms showing the percentage of time spent (A) or of distance travelled (C) by drones according to 15° sectors, with the odour quadrant being represented on the upper left (grey area). Light grey bars represent the 5 min before odour stimulation (‘before’), black bars represent the 5 min during stimulation (‘during’), and hence, dark grey bars show the overlap of the two phases. **B,D)** Histograms of the percentage of time spent (B), or of distance travelled (D) by drones in the odour quadrant (gray box) and on average in the three odourless quadrants (white box) before and during odour stimulation. (*): 0.05 < p < 0.1, Wilcoxon matched pairs tests.

**Fig 4:**
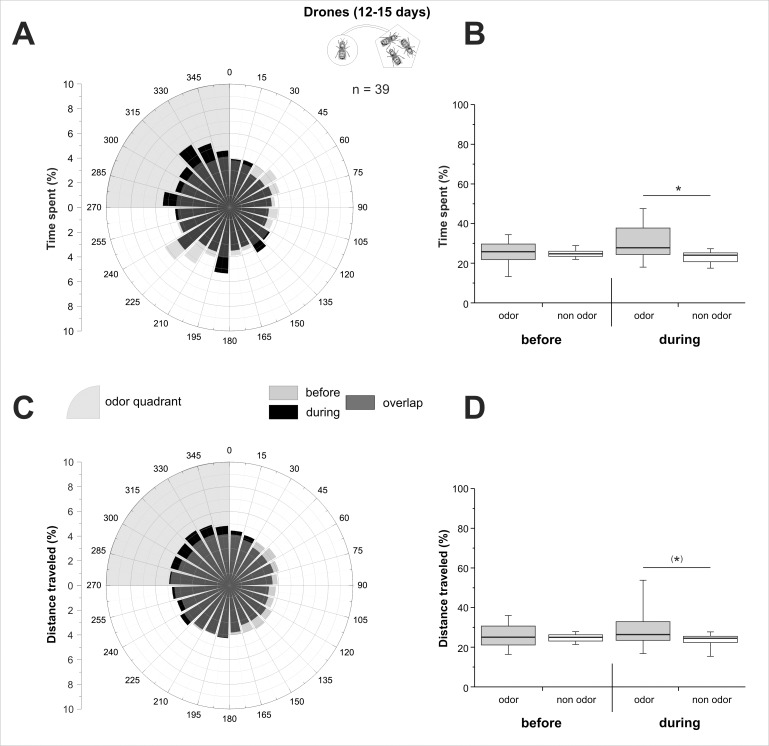
Olfactory attraction between 12–15 day-old drones. 12–15 day-old drones’ behaviour on the walking simulator, when stimulated with the odour bouquet of 10 same age drones. **A,C)** Circular histograms showing the percentage of time spent (A) or of distance travelled (C) by drones according to 15° sectors, with the odour quadrant being represented on the upper left (grey area). Light grey bars represent the 5 min before odour stimulation (‘before’), black bars represent the 5 min during stimulation (‘during’), and hence, dark grey bars show the overlap of the two phases. **B,D)** Histograms of the percentage of time spent (B), or of distance travelled (D) by drones in the odour quadrant (gray box) and on average in the three odourless quadrants (white box) before and during odour stimulation. *: p < 0.05; (*): 0.05 < p < 0.1; Wilcoxon matched pairs tests.

Ball movement data were acquired at 5 Hz frequency, so that drones' virtual position could be calculated every 200 ms, giving access to its virtual heading and the distance covered. In the figures, we represented the percentage of the time spent and of the distance travelled during each phase, either as a circular graph by 15° sectors (Figs 2A, 2C, 3A, 3C, 4A and 4C), or as a boxplot for the odour quadrant and the average of the 3 other quadrants (Figs 2B, 2D, 3B, 3D, 4B and 4D). To detect a significant orientation of bees toward the odour stimulus, time spent and distance travelled in the odour quadrant were compared to the average of the 3 other quadrants using Wilcoxon matched pairs tests. We compared the distance travelled by insects in the different experiments using a Kruskal-Wallis test. Pairwise comparisons were performed using Dunn’s test, which includes a correction for multiple comparisons. The Wilcoxon test was used to compare travelled distance between phases within each experiment. Graphs were plotted using OriginPro 8.5 (OriginLab, Northampton, MA, USA) and statistical analyses were performed using Statistica (StatSoft, Tulsa, OK, USA).

#### Chemical data

**Fig 5:**
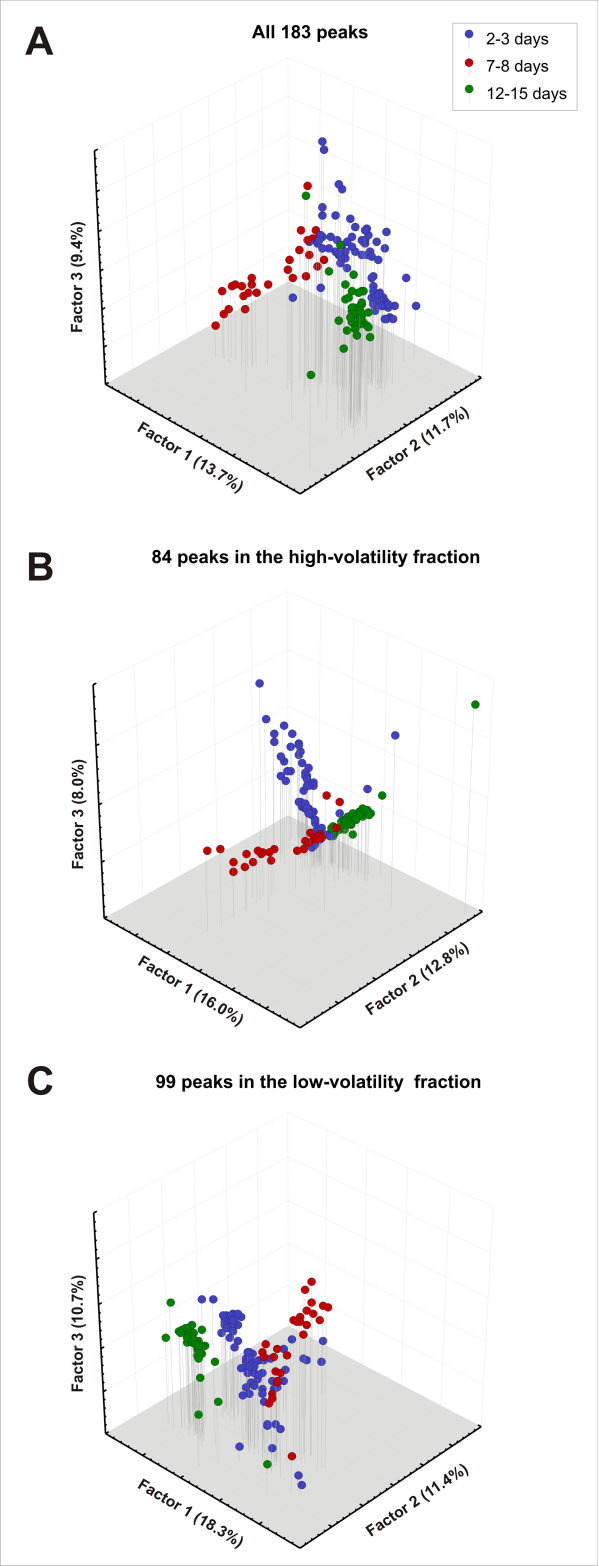
Age effect on drones’ chemical profile. The chemical composition of drone pentane extracts was analysed using gas-chromatography. The figure shows the representations of all individuals in the three age groups according to the first three factors of Principal Component Analyses (PCA) performed on (**A**) the 183 peaks of the whole chemical profiles, (**B**) only the 84 peaks of the high-volatility fraction, or (**C**) only the 99 peaks of the low-volatility fraction. The three PCAs show clear differences between the chemical profiles of the three age groups.

We aimed to examine differences in the chemical profiles of the different age groups, based on the relative proportions of chemical compounds found in their extracts. A total of 183 peaks were identified on the GC profiles of the different aged drones (S1A Fig). Within each individual, relative proportions of the different chemical compounds were calculated by dividing the area of each peak by the total sum of areas from the 183 peaks retained. In a first approach, we used the drones’ whole chemical profile (183 peaks) to evaluate possible differences between age groups. First, we performed Principal Component Analyses (PCA) with the SPAD 5.5 (Decisia) software (Fig 5). This analysis determines orthogonal axes (factors) of maximum variance in the data, and thus projects the data into a lower-dimensionality space formed of a subset of the highest-variance components. One of our key questions is whether drones’ chemical profile may contain *volatile* chemical cues indicating a drones’ age to other drones. We thus ran these analyses again on two subfractions of drones’ chemical profile. The high-volatility fraction consisted of the first 84 peaks of the profile, with retention times lower than that of Tricosane (n-C23) (S1B Fig). The low-volatility fraction consisted of the 99 peaks above this threshold (S1C Fig). The n-C23 threshold was chosen for several reasons: 1) cuticular hydrocarbons with chain lengths until this threshold can be found in insects’ volatile fraction [64]; 2) a previous account of drone cuticular profiles contained only molecules above this threshold [54]; 3) in terms of peak numbers, this threshold roughly segregated drones’ chemical profiles in two almost equal fractions. Two separate PCAs were thus performed with the low-volatility and with the high-volatility fractions (Fig 5B and 5C). To evaluate differences among the chemical profiles of the different age groups, we compared their coordinates on each of the first five factors, which together accounted for 45.7–53.1% of total variance. Kruskal-Wallis tests were used and followed, when significant by Dunn’s posthoc test. In the main figures, only the first three factors are represented (34.7–40.4% of total variance) (Fig 5).

**Fig 6:**
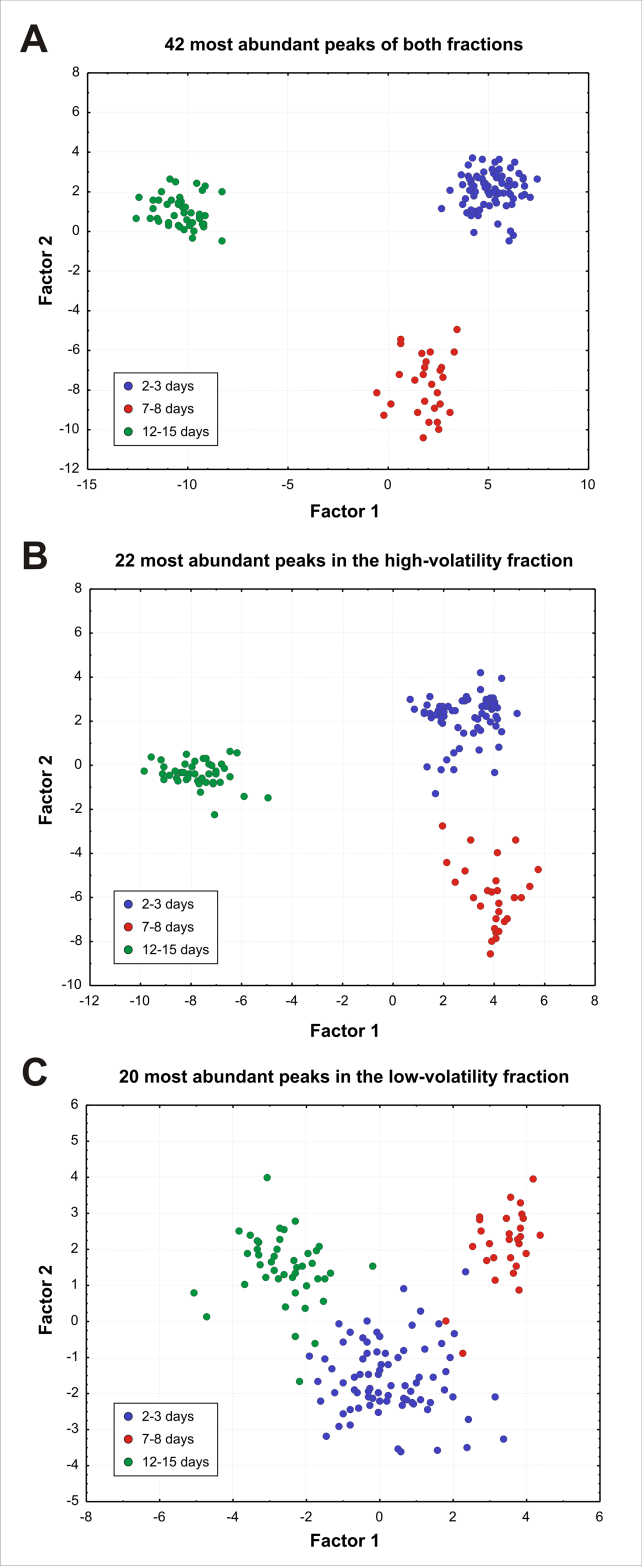
Age effect on drones’ chemical profile—most abundant compounds. Linear discriminant analyses performed on *abundant* compounds (defined as amounting to more than 1% within each fraction), (**A**) using the 42 abundant peaks from both fractions, (**B**) the 22 abundant peaks of the high-volatility fraction (**C**) the 20 abundant peaks of the low-volatility fraction. Discriminant analyses clearly segregated the drones depending on their age in all cases. Separation was slightly less marked in the case of the low-volatility fraction.

To further demonstrate the separation of the chemical profiles of the different age groups, discriminant analyses (DA) were performed. Due to the inherent limitations of this analysis with respect to the ratio between the numbers of cases and variables [65,66], only a subset of the peaks could be used. Therefore, within each fraction, only compounds with a mean abundance above 1% of the total amount of chemicals in this fraction were included. This threshold selected 22 peaks for the high-volatility fraction and 20 peaks for the low-volatility fraction. We thus performed a first DA with all the 42 peaks and two separate DAs with the 22 high-volatility and the 20 low-volatility compounds respectively (Fig 6A–6C). Prior to these analyses, percentage data were subjected to an arcsine transformation [67].

**Fig 7:**
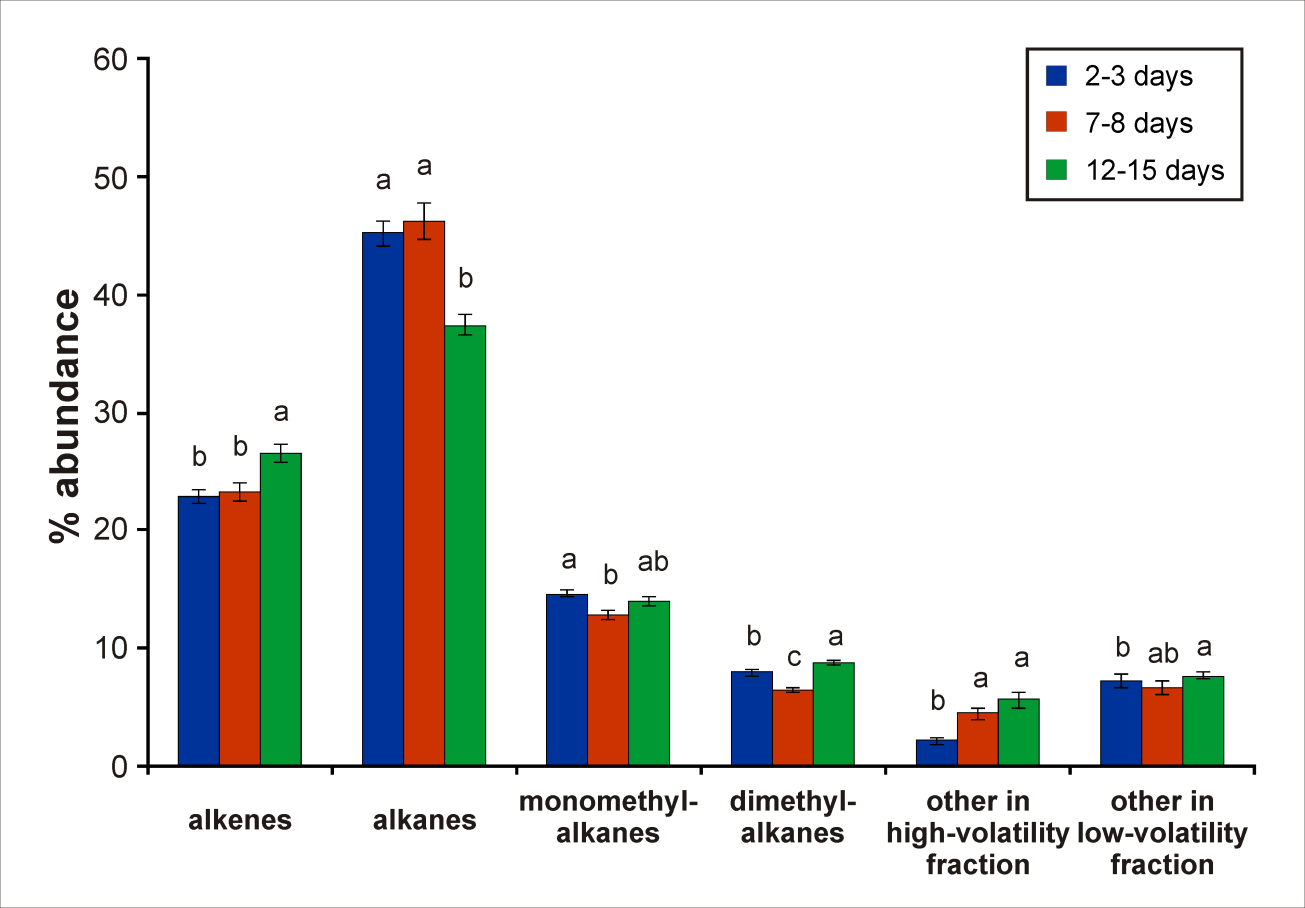
Mean proportions (± SEM) of five classes of compounds in drones according to age. Considering typical cuticular hydrocarbons (mostly from the low-volatility fraction), mature drones (12–15 days old) had significantly higher levels of alkenes and dimethyl alkanes but lower levels of straight chain alkanes compared to younger drones (2–3 and 7–8 days old). Apart from these, 2–3 day-old drones had significantly lower levels of highly-volatile compounds than older drones (7–8 and 12–15 days old). Letters indicate significant differences between groups of age (Kruskal-Wallis test followed by Dunn’s post-hoc test).

Lastly, we asked which types of molecules may be good candidates for age-dependent drone cues. First, we sorted molecules in different groups sharing functional characteristics, i.e. alkanes, monomethylalkanes, dimethylalkanes, alkenes (mono and bi-unsaturated). Other compounds (including non-identified ones) were simply grouped as belonging to the high-volatility or the low-volatility fraction. Then we compared the proportions of each compound type between age groups using Kruskal-Wallis tests followed by Dunn tests for multiple comparisons (Fig 7). For highlighting interesting molecules in the whole dataset, the same approach was applied to each of the 183 peaks. The results of these tests are reported in a comprehensive table (S1 Table). All statistical analyses were performed using Statistica (Statsoft, Tulsa, OK, USA).

## Results

### Distance travelled by drones in the walking simulator

To investigate the role of maturation in drone’s mutual attraction, we studied three groups of age, young drones (2–3 days old), middle-aged drones (7–8 days old) and adult drones (12–15 days old). All groups were active in the walking simulator, walking on and turning the ball. We observed however a significant heterogeneity among the distances travelled in 10 min by drones of the different age groups (Kruskal-Wallis test, H = 13.75, p = 0.0010; S2 Fig). The older drones (12–15 days old) travelled a significantly longer distance (3969 ± 621 mm) than younger drones (Dunn’s multiple comparisons: *2–3 vs 12–15 days old*, q = 2.68, p = 0.022; *7–8 vs 12–15 days old*, q = 3.64, p = 0.0008). The distance travelled by 2–3 days old (2740 ± 447 mm) and 7–8 day-old drones (1881 ± 228 mm) was not significantly different (q = 1.21, p = 0.67). In two of the groups, the travelled distance changed over time i.e. between the accommodation phase (‘before’) and the odour stimulation phase ('during'): in 2–3 and 7–8 day-old drones, the distance decreased over time (Wilcoxon matched pairs test, Z = 2.70, p = 0.007 and Z = 4.18, p = 0.0001 respectively). The distance travelled by 12–15 day-old drones remained stable throughout the experiment (Z = 1.70, p = 0.088).

### Maturation effect on drone’s mutual attraction

To quantify odour attraction, we measured the time spent and the distance travelled by drones in the odour quadrant relative to the three non-odour quadrants.

#### 2–3 day-old drones

The young drones (2–3 days old) showed a homogeneous circular repartition in the four different quadrants, both for the time spent (Fig 2A) and for the distance travelled (Fig 2C) in the two phases of the experiment. Accordingly, the time spent (Fig 2B) and the distance travelled (Fig 2D) by 2–3 day-old drones did not differ between the odour quadrant and the non-odour quadrants, neither during the accommodation phase (‘before’, Wilcoxon test, Z_time_ = 0.09, p_time_ = 0.93; Z_distance_ = 0.08, p_distance_ = 0.94) nor during the stimulation phase (‘during', Z_time_ = 0.78, p_time_ = 0.43; Z_distance_ = 0.28, p_distance_ = 0.78). We conclude that the odour bouquet of 10 living 2–3 day-old drones did not induce any significant change in same age drone’s behaviour.

#### 7–8 day-old drones

The 7–8 day-old drones showed apparently different circular repartitions in the four different quadrants for the time spent (Fig 3A) and for the distance travelled (Fig 3C) between the two experimental phases. In the accommodation phase (‘before’), the time spent (Fig 3A) as well as the distance travelled (Fig 3C) by the 7–8 days drones were homogenous in all directions. Accordingly, they showed no difference in the time spent (Fig 3B) or in the distance travelled (Fig 3D) between odour and non-odour quadrants (Z_time_ = 0.28, p_time_ = 0.78 and Z_distance_ = 1.09, p_distance_ = 0.27). However, their circular orientation appeared heterogeneous during odor presentation (Fig 3A and 3C), with remarkable incursions in the odour quadrant (upper left quadrant). This tendency did however not reach the statistical threshold for the time spent in the odour relative to the non-odour quadrants (Z_time_ = 1.04, p_time_ = 0.30) but was only near-significant for the travelled distance (Z_distance_ = 1.72, p_distance_ = 0.085). This experiment suggests that the odour bouquet from 7–8 day-old drones was not strongly attractive to same age drones.

#### 12–15 day-old drones

Older drones (12–15 days old) showed an homogeneous circular repartition in the four different quadrants during the accommodation phase (‘before’), both for the time spent (Fig 4A) and the distance travelled (Fig 4C). Accordingly, these two measures (Fig 4B and 4D respectively) were not significantly different between odour and non-odour quadrants in this phase (Z_time_ = 0.43, p_time_ = 0.67; Z_distance_ = 0.57, p_distance_ = 0.57). In the odour stimulation phase (Fig 4A, ‘during’), however, the behaviour of 12–15 day-old drones changed and they started to orient toward the bouquet of 10 adult drones (upper left quadrant). Accordingly, 12–15 day-old drones spent significantly more time in the odour quadrant than the non-odour quadrants (Fig 4B, Z_time_ = 2.40, p_time_ = 0.016). The effect on the travelled distance (Fig 4C) was more subtle, being only near-significantly higher in the odour quadrant compared to the non-odour quadrants (Z_distance_ = 1.70, p_distance_ = 0.089). These results suggest that 12–15 day-old drones are attracted to volatile molecules produced by same-age drones. We conclude that when drones have access to the effluent bouquet from a group of 10 drones, they display an age-dependent olfactory attraction, which culminates in older drones (12–15 days old).

#### Age-related changes in drones’ chemical profile

The behavioural experiments showed an olfactory attraction between mature drones (12–15 days old) but not between younger drones (2–3 and 7–8 days old). We searched for a possible chemical basis for this behavioural effect. We thus evaluated the effect of drone maturation on their chemical profile. Pentane extracts analyzed with gas chromatography and mass spectrometry revealed a total of 183 peaks corresponding to individual chemical compounds (see S1 Fig for a typical drone profile). The extract could be divided into a low- and a high-volatility fraction (see Methods). A global principal component analysis (PCA) showed that the drones’ complete chemical profile was indeed different in the three age groups (Fig 5A). The first five factors represented 45.7% of overall variance. On four of these factors, a significant heterogeneity was observed among the coordinates of the three groups (Factors 1,2,4 and 5; Kruskal-Wallis test, H > 25.8, p < 0.001; S3 Fig, left column). In particular, on Factors 1, 4 and 5, the coordinates of 12–15 day-old drones were significantly different from those of the two other groups (Dunn test, p < 0.05).

The same conclusions appeared when only the high-volatility (Fig 5B) or only the low-volatility fractions (Fig 5C) were considered. For the high-volatility fraction, among the first five factors (49.0% variance), Factors 1,2,4 and 5 showed a significant heterogeneity among age groups (Kruskal-Wallis test, H > 16.0, p < 0.001; S3 Fig, middle column). Here, Factors 1,2 and 5 showed a significant difference between the coordinates of 12–15 day-old drones and those of the two other groups (Dunn test, p < 0.05). Factor 1 was a clear age group, its coordinates varying progressively with drones’ age and all three groups’ coordinates being different from each other (Dunn test, p < 0.05). For the low-volatility fraction, among the first five factors (53.1% variance), Factors 1,2,3 and 4 displayed a significant heterogeneity among age groups (Kruskal-Wallis test, H > 47.0, p < 0.001; S3 Fig, right column). Here, Factors 1,2 and 4 showed a significant difference between the coordinates of 12–15 day-old drones and those of the two other groups (Dunn test, p < 0.05). Factor 4 was a clear age group, its coordinates varying progressively with drones’ age and all three groups’ coordinates being different from each other (Dunn test, p < 0.05).

The ability of drones’ chemical profiles to be separated according to the drones’ age was further demonstrated by linear discriminant analyses (Fig 6A–6C). These analyses, which emphasize differences among groups, clearly segregated the drones according to their age, even with only two factors. This result was very clear when focusing on the 22 most abundant compounds (> 1%) of the high-volatility fraction (Fig 6B, Wilk’s lambda = 0.0037, F_32,266_ = 129.3, p < 0.001, all pairwise comparisons significant p < 0.01), but slightly less, although still significant, when using the 20 most abundant compounds of the low-volatility fraction (Fig 6C, Wilk’s lambda = 0.050, F_36,262_ = 25.4, p < 0.001, all pairwise comparisons significant p < 0.01). We thus conclude that the drones’ chemical profile changes with their age and that this maturation affects both the high-volatility and the low-volatility fractions.

We next aimed to determine which categories of compounds changed with age. We thus compared the three age groups depending on chemical characteristics of identified cuticular compounds (Fig 7). In low-volatility molecules, 12–15 day-old drones had significantly higher levels of alkenes and dimethyl alkanes, and lower levels of straight chain alkanes compared to younger drones (Kruskal-Wallis test, H > 25.5, p < 0.001, posthoc Dunn tests, p < 0.01). Most interestingly, high-volatility compounds increased progressively between 2–3 day-old and older drones (7–8 and 12–15 days old) (Kruskal-Wallis test, H = 58.9, p < 0.001, posthoc Dunn tests, p < 0.001). Among those, some may play a role as a cue in drone mutual attraction. To pinpoint possible candidates, we systematically compared the quantities of the 183 compounds in the three age groups (S1 Table). Mature (12–15 days old) drones had significantly higher levels for 36 compounds compared to younger drones (2–3 and 7–8 days old), 15 compounds in the high-volatility fraction and 21 compounds in the low-volatility fraction (Kruskal-Wallis test, H > 25.0, p < 0.001, posthoc Dunn tests, p < 0.05) (S1 Table). In the 15 volatile compounds that were more abundant in mature drones, 9 compounds were found in at least 89% of 12–15 day-old drones and could be putative attraction pheromone in mature drones. They are highlighted in grey in S1 Table.

## Discussion

In this study, we analysed the effect of drone’s age on their mutual olfactory attraction and on their chemical profiles. Using a walking simulator, we show that young drones (2–3 and 7–8 days) do not show mutual olfactory attraction whereas older drones (12–15 days) do, as observed on flight-ready drones in an earlier study [44]. In parallel, we found a variation according to age in the drones’ chemical profiles analysed by GC/MS, both for low-volatility and high-volatility fractions. These data suggest that drones’ aging, which correlates with their sexual maturation, is accompanied by a modification in their volatile emissions, which may be involved in the observed olfactory attraction between mature drones.

First, we noticed an age effect on drone’s propensity to walk on the tread ball of the walking simulator. We found that older drones (12–15 days old) travelled a longer distance than younger ones (2–3 and 7–8 days old). Interestingly, the locomotor activity found here for 12–15 day-old drones was similar to that of drones captured when flying out of the hive in a previous study [44]. In addition, older drones (12–15 days) did not show any significant decrease in the travelled distance between the two experimental phases, contrary to younger drones (2–3 and 7–8 days old). This can be interpreted in two non-exclusive ways: first, it may indicate that older drones, after a long period of gathering energy within the hive, are more robust and possess better endurance than younger ones. Second, it is also possible that after detecting an attractive substance (the effluent from other drones, see below), older drones are more highly motivated for walking on the ball, searching for the substance source. We conclude that the age of drones, and accordingly their sexual maturity, has an effect on their locomotor activity with an increase until the age of 12 days.

Concerning drones’ olfactory orientation, our results show no olfactory attraction in young drones (2–3 days old). Since in our experiments, stimulation drones and focal drones (i.e. on the ball) have the same age, this result may involve both emitter and receiver effects. On the one hand, one can assume that at that age, drones are not yet mature enough to produce volatiles than are attractive to other males. On the other hand, their olfactory system may not have matured enough for detecting/responding to such substances. While 12–15 day-old drones preferred the odour from other drones, no significant attraction was observed for 7–8 day-old drones. We noticed however that during the stimulation phase, this age-group displayed a higher inter-individual variability compared to both younger and older drones (compare box and whisker sizes in Fig 3B and 3D to those in Figs 2B, 2D, 4B and 4D). Many physiological and behavioural changes occur during this period of a drone’s life, but they may not take place at exactly the same time in all individuals. Indeed, considerable variation can be found in the literature regarding two usual measures of drones’ sexual maturity, the first flight out of the hive (7 to 9 days [55–58]) and the highest numbers of spermatozoa in the seminal vesicles (8 to 12 days [18,60]). It is thus possible that within a group of 7–8 day-old drones, some are already mature and are attracted to other males’ odour, while others are still undergoing maturation and do not display any such attraction yet. Since stimulation males are in groups of ten, a few mature males in this group may be sufficient for providing an attractive odour to the freshly mature focal drones. To illustrate these thoughts, in the 7–8 day-old group, 25% of the drones spent more than 35% of their time in the odour quadrant, a similar proportion as in the 12–15 day-old group (18%). The same was also true for the travelled distance, with 23% of 7–8 days and 21% of 12–15 day-old drones running more than 35% of the total distance in the odour quadrant. In any case, by 12 days of age, drones are universally considered sexually mature, engaging in real mating flights and displaying a maximal abundance of mature semen [22,68,69]. We conclude that drone mutual attraction by means of volatile substances develops with age, most likely through their sexual maturation, a process that is achieved after 12 days of age, but possibly starts for some individuals as early as the 7–8^th^ day.

Although our initial motivation for performing these experiments was related to drones’ role in mating behaviour, our results must also be interpreted according to in-hive processes. In our set up, the drones were walking, not flying, and since we wanted to test olfactory attraction in the absence of visual cues, the experiments were performed in the dark. Thus, the experimental situation resembles the context of drone orientation within the hive. During their first days of life, the drones are located on the central combs where temperature is the warmest and they can be fed by workers [48–51]. On the other hand, mature drones are mostly found on the periphery of the combs, feeding themselves directly from the stores [48]. In both locations, the drones do not appear randomly distributed but tend to form clusters [49,51]. These aggregates could be related to subtle local cues like small differences in food supply and/or temperature, but they may also involve chemical cues. For young drones (a few days old), our results allow discarding a role of volatile substances since our focal animals never come in contact with stimulation animals but only receive their effluent via an airflow. However, non-volatile chemical cues may be involved. Interestingly, a theoretical model already suggested the existence of non-volatile substances produced by the drones, which may play a role in the regulation of drone production in the colony [70]. Such a cue is as yet unknown but could be present in the non-volatile fraction of our samples. Concerning older drones’ aggregation, volatile substances may play a role as suggested by our behavioural results.

Because we wanted to test olfactory attraction *per se*, we could not provide the drones with the level of multimodal sensory information they receive in a natural mating context. This does not preclude however the possibility that the drone olfactory attraction we observed may actually take place during mating flights. We find it indeed remarkable that mutual olfactory attraction in drones correlates with their sexual maturity, strengthening the hypothesis that this mutual attraction may play a role in bees’ mating behaviour. Taking into account previous demonstration of drone mutual attraction both in a walking simulator [44] and in free-flying conditions [42,43], one may propose that drone-emitted volatiles contribute to the formation of drone congregations first by accelerating drone aggregation and then by stabilizing the drone cloud.

Our behavioural results imply that drones’ volatiles emissions and/or their olfactory circuits change with age. Our chemical analyses confirmed the first part of this proposal by revealing a difference in drones’ chemical profile according to age. The change in low-volatility molecules (which includes cuticular hydrocarbons) is consistent with that described in a previous study [54]. We observed that mature drones contained higher levels of dimethyl alkanes and alkenes but lower levels of alkanes relative to younger drones. Such differences in low-volatility molecules could play an important role in the hive for the recognition and the differential treatments granted to young vs older drones by workers [48,51,71]. Typically, workers feed drones only when they are young (until 7–8 days of age [48]). They communicate with young drones through vibration signals, inducing more activity from the drones, which receive in turn more intense feeding and grooming [51,71]. Then, at the beginning of autumn, young drones are still nursed by workers while older drones are ejected from the colony [48]. Our analyses also show that the high-volatility fraction of the drone chemical profile (eluting before n-C23) varies with age, possibly explaining the age-specific olfactory attraction we observed in our behavioural data. In the context of mating, volatile stimuli seem more adequate than low-volatility compounds for supporting the formation of a drone congregation high in the air. This putative substance needs to attract other drones (but also virgin queens, see [45]) from long distances, given that drones travel up to 15 km to find a congregation area [72]. Our results thus point to the production of one or more attractive volatile molecules by sexually mature drones, which do not occur, or in too small quantities in younger drones to support mutual attraction. Mature (12–15 days old) drones displayed significantly higher levels of specific compounds relative to younger drones (2–3 and 7–8 days old) (S1 Table). Among these, 9 compounds were present in almost all 12–15 day-old drones and would be especially interesting to further study in walking simulator experiments.

The age effect we observed on drone mutual attraction could also be due to a maturation of their olfactory system. The drone olfactory system is specially adapted for the detection and processing of mating relevant olfactory cues [32]. The drone antenna is twice as large as that of the worker and carries ~7 times as many sensilla placodea [73,74]. Within these, a type of olfactory sensory neuron specifically responding to 9-ODA has been observed [75,76]. Electroantennogram (EAG) recordings showed that the drone antenna is much more sensitive to 9-ODA than the worker antenna [77,78]. The first olfactory relay of the drone brain, the antennal lobe, contains four hypertrophied glomeruli (termed macroglomeruli, MG [79,80]), one of which responds specifically to 9-ODA [81]. Similarly, transcriptomic analyses show that four olfactory receptor proteins (ORs) are overexpressed in the drone antenna compared to the worker antenna [82]. One of these four ORs, AmOR11 detects 9-ODA [82]. To this day, the role of the three remaining MGs and of the three remaining overexpressed ORs is unknown. The question thus arises if one or more are involved in the detection/processing of drone-produced odour cues.

The drone olfactory system, like that of workers, undergoes a maturation process that continues during the first days after adult emergence [83]. Several studies evaluated the progression of EAG responses to queen volatiles at different ages with slightly contradictory results [77,84,85]. While Skirkevieiene and Skirkevieius [84] observed a steady increase in EAGs to a queen extract in the first 8 days of a drone’s life, Vetter and Visscher [77] describe a general decrease of EAGs in the course of 40 days (an old age for drones), while Villar et al., [85] found lower responses to 9-ODA at 14 days than at 4 days. From these observations, one may gather that the sensitivity of the drone antenna increases in the first days after emergence, as is well established in workers [86–88], shows a plateau around 8 days but undergoes a slow decrease after that. We thus conclude that in addition to the demonstrated changes in drones’ volatile emissions, changes in their olfactory sensitivity may also be involved in the age-dependent mutual attraction we observed.

In conclusion, this study showed that drone mutual olfactory attraction is age dependent and coincides with the age when drones are considered sexually mature. At the same time, drones’ chemical profile changes with age, including its volatile fraction. While olfactory attraction between older males may relate to drone clustering within the hive, our results could also indicate a role of a volatile substance in honey bee mating, possibly in the establishment and stabilization of drone congregations. The few molecules whose quantity clearly increases with drones’ age could constitute interesting candidates for a putative drone aggregation pheromone.

## Supporting information

S1 Fig Chemical profile of mature drones. Typical profile of a 12–15 day-old drone obtained by gas chromatography. The figure shows (**A**) the whole extract, (**B**) the high-volatility fraction, and (**C**) the low-volatility fraction. The n-C23 peak segregating both fractions is indicated in A and C. The Y axis shows the abundance of the compounds (in arbitrary units) and the X axis the retention time.
(TIF)

S2 Fig Total distance travelled by drones according to age. Distance travelled on the walking simulator, ‘before’ (5 min) and ‘during’ odour stimulation (5min), for each group of drones: 2–3, 7–8 and 12–15 days old. White bars represent the phase ‘before’ odour stimulation, whereas grey bars represent the phase ‘during’ odour stimulation. Letters indicate significant differences between groups of age (Kruskal-Wallis test, followed by Dunn’s post-hoc test). Asterisks reveal significant differences between ‘before’ and ‘during’ phases (Wilcoxon matched pairs tests, ** p < 0.01, *** p < 0.001).
(TIF)

S3 Fig Comparison of drones’ chemical profiles with the first five factors of principal component analyses. The first five factors together accounted for 45.7% of total variance for the whole profile, 49.0% for the high-volatility fraction, and 53.1% for the low-volatility fraction. Heterogeneity was observed among the coordinates of the three groups of age. In particular, the coordinates of 12–15 day-old drones were significantly different from those of the two other groups for the whole profile (factors 1, 4 and 5), for the high-volatility fraction (factors 1, 2 and 5) and for the low-volatility fraction (factors 1,2 and 4). The results of each Kruskal-Wallis test is indicated on the upper right of each panel: KW ***: p < 0.001; NS: non-significant. Letters indicate significant differences between groups of age (Dunn’s post-hoc test).
(TIF)

S1 Table List and relative proportions of the compounds found in drone extracts at different ages. Relative compound concentrations are provided as mean ± SEM. Tentative identifications from mass spectra were made using NIST Mass spectral search 2.2. The n-C23 threshold (selected delimitation between high-volatility and low-volatility fractions) is shown in red. Compounds with a gray background are significantly more abundant in 12–15 day-old drones and were found in > 89% of the drones. For each line, letters indicate significant differences in Dunn’s post-hoc tests when the Kruskal-Wallis test was significant.
(PDF)
