# Supplementary figures and images for: Age-specific olfactory attraction between Western honey bee drones (Apis mellifera) and its chemical basis
Source: PLoS One. 2017 Oct 4;12(10):e0185949. doi: 10.1371/journal.pone.0185949 (PMC5627955; doi:10.1371/journal.pone.0185949)

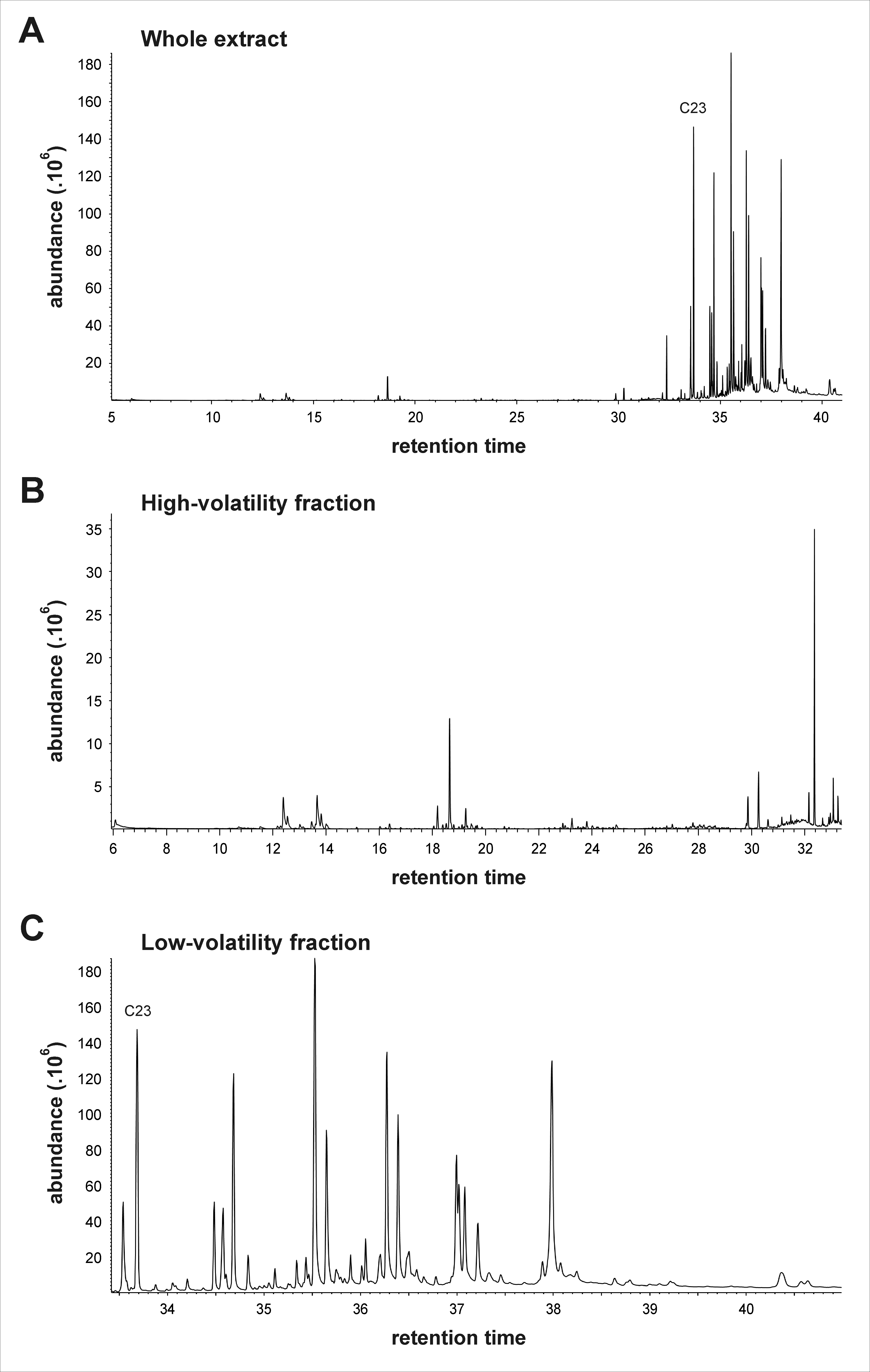

Supplement: S1 Fig — Typical profile of a 12–15 day-old drone obtained by gas chromatography. The figure shows (A) the whole extract, (B) the high-volatility fraction, and (C) the low-volatility fraction. The n-C23 peak segregating both fractions is indicated in A and C. The Y axis shows the abundance of the compounds (in arbitrary units) and the X axis the retention time. (TIF) [file pone.0185949.s001.tif]

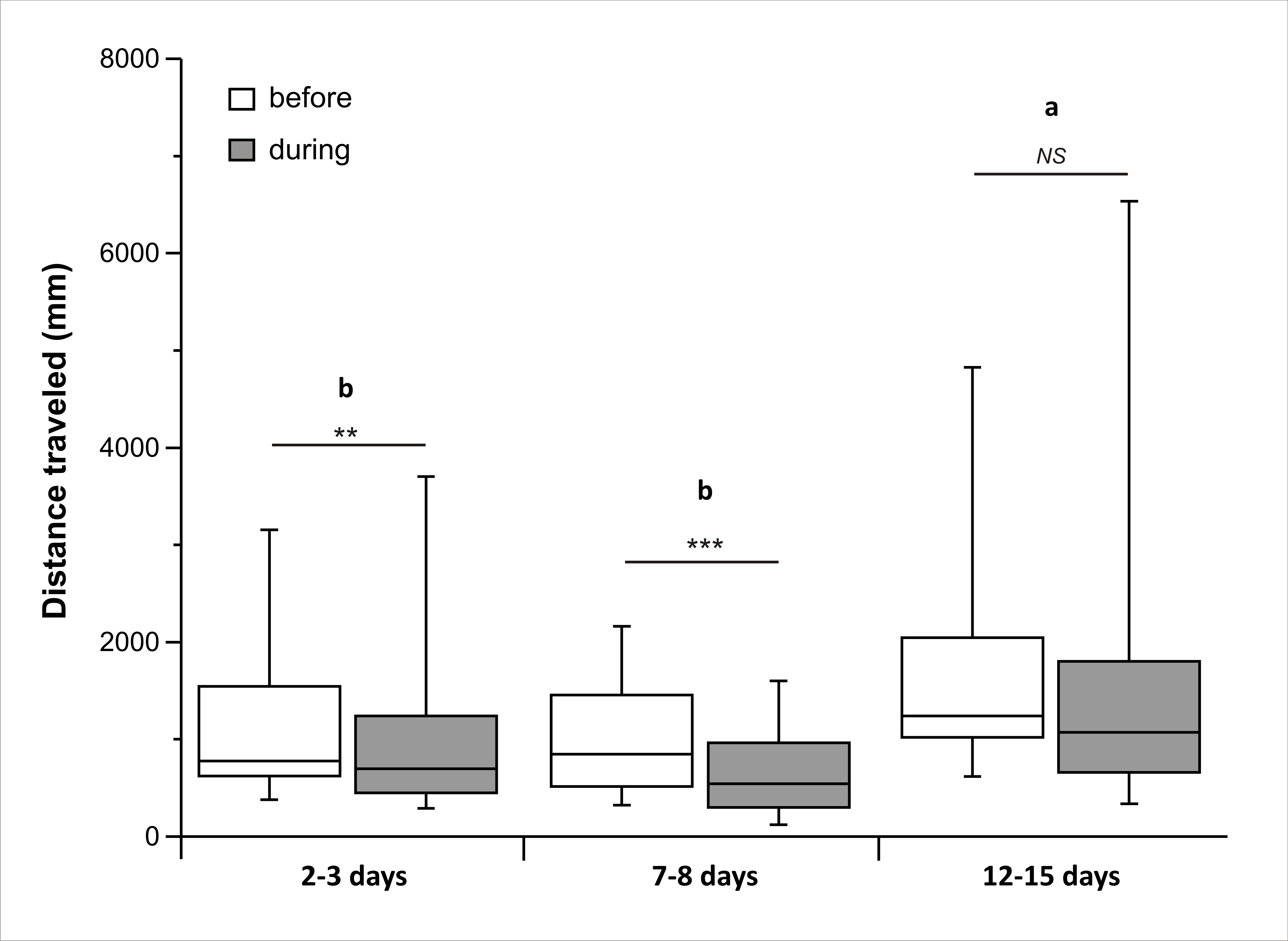

Supplement: S2 Fig — Distance travelled on the walking simulator, ‘before’ (5 min) and ‘during’ odour stimulation (5min), for each group of drones: 2–3, 7–8 and 12–15 days old. White bars represent the phase ‘before’ odour stimulation, whereas grey bars represent the phase ‘during’ odour stimulation. Letters indicate significant differences between groups of age (Kruskal-Wallis test, followed by Dunn’s post-hoc test). Asterisks reveal significant differences between ‘before’ and ‘during’ phases (Wilcoxon matched pairs tests, ** p < 0.01, *** p < 0.001). (TIF) [file pone.0185949.s002.tif]

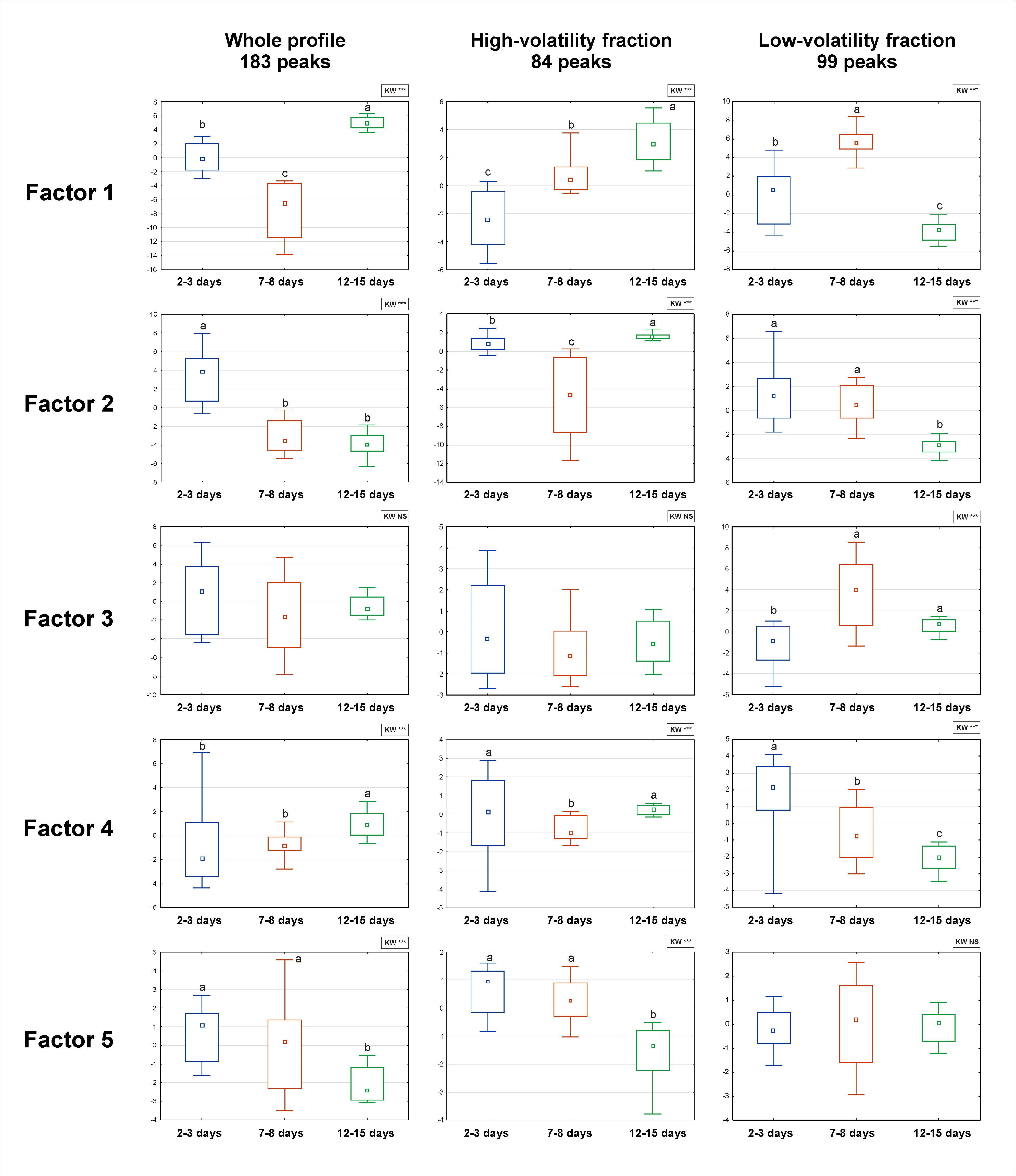

Supplement: S3 Fig — The first five factors together accounted for 45.7% of total variance for the whole profile, 49.0% for the high-volatility fraction, and 53.1% for the low-volatility fraction. Heterogeneity was observed among the coordinates of the three groups of age. In particular, the coordinates of 12–15 day-old drones were significantly different from those of the two other groups for the whole profile (factors 1, 4 and 5), for the high-volatility fraction (factors 1, 2 and 5) and for the low-volatility fraction (factors 1,2 and 4). The results of each Kruskal-Wallis test is indicated on the upper right of each panel: KW ***: p < 0.001; NS: non-significant. Letters indicate significant differences between groups of age (Dunn’s post-hoc test). (TIF) [file pone.0185949.s003.tif]
