# Supplementary material for: Age-specific olfactory attraction between Western honey bee drones (Apis mellifera) and its chemical basis
Source: PLoS One. 2017 Oct 4;12(10):e0185949. doi: 10.1371/journal.pone.0185949 (PMC5627955; doi:10.1371/journal.pone.0185949)
Supplement: S1 Table — Relative compound concentrations are provided as mean ± SEM. Tentative identifications from mass spectra were made using NIST Mass spectral search 2.2. The n-C23 threshold (selected delimitation between high-volatility and low-volatility fractions) is shown in red. Compounds with a gray background are significantly more abundant in 12–15 day-old drones and were found in > 89% of the drones. For each line, letters indicate significant differences in Dunn’s post-hoc tests when the Kruskal-Wallis test was significant. (PDF) [file pone.0185949.s004.pdf]

Peak No	Retention Time	Identification	Drones 2-3 days	Drones 7-8 days	Drones 12-15 days	Kruskal-Wallis test	
			mean ± SEM	mean ± SEM	mean ± SEM	H	p
1	6.083	C9	0.009 ± 0.002	None	None	12.844	0.001
2	11.168	2-hydroxyethyl methacrylate (74%)	0.553 ± 0.204 (c)	1.119 ± 0.243 (b)	2.332 ± 0.400 (a)	68.026	0.000
3	12.446	unidentified hydrocarbon	0.132 ± 0.025 (a)	0.019 ± 0.008 (ab)	None (b)	18.921	0.000
4	12.549	unidentified hydrocarbon	0.055 ± 0.010 (a)	None (ab)	None (b)	25.579	0.000
5	13.459	unidentified hydrocarbon	0.008 ± 0.003	None	None	7.641	0.021
6	13.606	unidentified hydrocarbon	0.144 ± 0.026 (a)	None (ab)	None (b)	25.579	0.000
7	13.716	unidentified hydrocarbon	None	0.024 ± 0.008	0.008 ± 0.003	21.786	0.000
8	13.819	unidentified hydrocarbon	0.053 ± 0.010 (a)	None (ab)	None (b)	25.579	0.000
9	16.012	C12:1	0.001 ± 0.000	None	None	2.771	0.250
10	16.109	methylsalicylate (93%)	None (b)	None (b)	0.175 ± 0.034 (a)	71.593	0.000
11	16.380	Decanal (58%)	0.004 ± 0.001 (b)	None (b)	0.079 ± 0.004 (a)	120.713	0.000
12	18.195	unidentified hydrocarbon	0.045 ± 0.008 (a)	None (ab)	None (b)	25.579	0.000
13	18.236	unidentified hydrocarbon	None	0.009 ± 0.005	None	13.355	0.001
14	18.803	unidentified hydrocarbon	0.007 ± 0.005	0.009 ± 0.006	0.014 ± 0.011	1.574	0.455
15	19.256	unidentified hydrocarbon	0.032 ± 0.006 (a)	0.005 ± 0.003 (ab)	None (b)	17.751	0.000
16	19.465	unidentified hydrocarbon	0.001 ± 0.001	None	None	1.835	0.399
17	19.687	unidentified hydrocarbon	0.001 ± 0.001	0.012 ± 0.007	0.002 ± 0.001	4.955	0.083
18	20.652	beta-bourbonene (74%)	Trace	None	None	1.835	0.399
19	20.686	C14:1	Trace	None	None	1.835	0.399
20	22.909	unidentified hydrocarbon	0.005 ± 0.002	None	None	9.681	0.007
21	23.250	phenol, 2,4-bis(1,1 dimethylethyl)(58%)	0.060 ± 0.012 (a)	0.028 ± 0.013 (b)	0.012 ± 0.002 (b)	27.887	0.000
22	23.288	butylated hydrotoluene (64%)	0.007 ± 0.005	0.013 ± 0.009	0.082 ± 0.035	15.682	0.000
23	23.559	delta-cadinene (23%)	0.002 ± 0.001	None	None	2.771	0.250
24	23.811	unidentified hydrocarbon	0.012 ± 0.003 (b)	None (b)	0.985 ± 0.168 (a)	118.081	0.000
25	24.002	unidentified hydrocarbon	0.001 ± 0.000	None	None	1.835	0.399
26	24.182	bisabolene epoxide (34%)	0.001 ± 0.000	None	None	1.835	0.399
27	24.672	spathulenol (59%)	0.002 ± 0.001	None	None	2.771	0.250
28	24.797	caryophyllene oxyde (57%)	0.001 ± 0.001	None	None	1.835	0.399
29	26.814	C17	0.004 ± 0.001	None	None	16.135	0.000
30	27.092	unidentified hydrocarbon	0.018 ± 0.003 (b)	0.123 ± 0.023 (a)	0.001 ± 0.001 (c)	33.695	0.000
31	27.797	unidentified hydrocarbon	0.020 ± 0.003 (a)	None (b)	None (b)	37.469	0.000
32	27.868	unidentified hydrocarbon	Trace (b)	0.143 ± 0.025 (a)	0.001 ± 0.001 (b)	72.458	0.000
33	28.163	unidentified hydrocarbon	None (b)	0.031 ± 0.008 (a)	None (b)	46.66.20	0.000
34	28.212	unidentified hydrocarbon	0.001 ± 0.001	None	0.005 ± 0.004	2.243	0.325
35	28.365	benzenesulfonamide, N-butyl- (61%)	Trace (b)	None (b)	0.042 ± 0.011 (a)	48.726	0.000
36	28.501	unidentified hydrocarbon	None	0.002 ± 0.002	0.003 ± 0.002	5.156	0.075
37	28.642	unidentified hydrocarbon	None	0.005 ± 0.005	0.001 ± 0.001	2.442	0.295
38	29.187	unidentified	0.136 ± 0.064 (b)	0.260 ± 0.044 (a)	0.001 ± 0.001 (b)	82.171	0.000
39	29.867	C19:1	0.107 ± 0.010 (b)	0.005 ± 0.003 (a)	None (a)	59.292	0.000
40	29.890	unidentified	None	None	0.002 ± 0.001	9.924	0.007
41	29.941	C19:1	0.038 ± 0.007 (b)	0.096 ± 0.012 (a)	0.049 ± 0.004 (a)	23.014	0.000
42	30.324	C19	0.270 ± 0.014 (a)	0.208 ± 0.011 (a)	0.107 ± 0.007 (b)	67.197	0.000
43	30.679	7,9-di-tert-butyl-1-oxaspiro(4,5) deca-6,9-diene-2,8-dione (95%)	0.084 ± 0.010 (b)	0.556 ± 0.070 (a)	0.074 ± 0.004 (b)	53.087	0.000
44	30.733	unidentified hydrocarbon	0.001 ± 0.001 (b)	0.019 ± 0.005 (a)	None (b)	28.738	0.000
45	31.007	unidentified hydrocarbon	0.004 ± 0.002	None	None	6.641	0.036
46	31.065	unidentified hydrocarbon	0.002 ± 0.001	0.012 ± 0.004	0.001 ± 0.001	13.853	0.001
47	31.143	unidentified hydrocarbon	0.057 ± 0.007 (a)	0.010 ± 0.004 (b)	None (b)	54.400	0.000
48	31.185	unidentified hydrocarbon	0.008 ± 0.002 (c)	0.290 ± 0.035 (a)	0.028 ± 0.003 (b)	90.359	0.000
49	31.272	unidentified hydrocarbon	0.010 ± 0.003 (b)	0.040 ± 0.009 (a)	0.004 ± 0.002 (b)	18.650	0.000
50	31.311	unidentified hydrocarbon	None	None	0.006 ± 0.003	9.924	0.007
51	31.420	unidentified hydrocarbon	0.007 ± 0.003	0.014 ± 0.008	0.023 ± 0.007	3.751	0.153
52	31.469	C20	0.031 ± 0.003 (a)	0.009 ± 0.005 (b)	0.037 ± 0.005 (a)	16.988	0.000
53	31.521	unidentified hydrocarbon	0.013 ± 0.003 (b)	0.061 ± 0.014 (a)	0.063 ± 0.006 (a)	53.624	0.000
54	31.547	unidentified	0.001 ± 0.001 (b)	None (b)	0.050 ± 0.008 (a)	68.652	0.000
55	31.590	unidentified	0.005 ± 0.002 (b)	0.087 ± 0.017 (a)	None (b)	59.739	0.000
56	31.649	unidentified	0.004 ± 0.002 (b)	0.055 ± 0.013 (a)	0.006 ± 0.003 (b)	25.856	0.000
57	31.752	unidentified	0.016 ± 0.003 (a)	0.021 ± 0.009 (ab)	0.006 ± 0.002 (b)	12.698	0.000
58	31.728	unidentified	None	None	0.005 ± 0.003	7.393	0.024
59	31.863	oxybenzone (95%)	None (b)	None (b)	0.074 ± 0.016 (a)	81.887	0.000
60	32.080	unidentified	0.004 ± 0.002 (b)	0.006 ± 0.003 (ab)	0.025 ± 0.010 (a)	26.105	0.000
61	32.160	unidentified	0.001 ± 0.001	0.003 ± 0.002	0.001 ± 0.001	2.719	0.256

Peak No	Retention Time	Identification	Drones 2-3 days	Drones 7-8 days	Drones 12-15 days	Kruskal-Wallis test	
			mean ± SEM	mean ± SEM	mean ± SEM	H	p
62	32.196	C21:1	0.134 ± 0.021 (b)	0.198 ± 0.037 (b)	0.454 ± 0.069 (a)	48.377	0.000
63	32.308	unidentified	0.013 ± 0.005 (b)	0.024 ± 0.012 (b)	0.118 ± 0.018 (a)	73.849	0.000
64	32.248	unidentified hydrocarbon	0.002 ± 0.001	0.002 ± 0.002	None	3.701	0.157
65	32.318	unidentified	0.006 ± 0.006	0.008 ± 0.008	None	1.640	0.440
66	32.400	C21	0.986 ± 0.035 (a)	0.811 ± 0.041 (b)	0.786 ± 0.029 (b)	16.813	0.000
67	32.484	unidentified hydrocarbon	0.007 ± 0.003 (b)	0.014 ± 0.007 (b)	0.028 ± 0.006 (a)	35.574	0.000
68	32.546	unidentified	0.038 ± 0.017 (b)	0.094 ± 0.031 (b)	0.196 ± 0.030 (a)	72.925	0.000
69	32.598	unidentified	0.026 ± 0.012 (b)	0.064 ± 0.023 (b)	0.221 ± 0.033 (a)	79.167	0.000
70	32.625	unidentified	None (b)	None (ab)	0.008 ± 0.002 (a)	31.420	0.000
71	32.708	unidentified hydrocarbon	0.012 ± 0.007 (b)	0.326 ± 0.040 (a)	0.157 ± 0.021 (a)	106.715	0.000
72	32.734	unidentified hydrocarbon	0.015 ± 0.008	None	None	17.262	0.000
73	32.762	unidentified hydrocarbon	0.006 ± 0.004 (b)	0.053 ± 0.011 (a)	None (b)	47.047	0.000
74	32.791	unidentified hydrocarbon	None (b)	0.027 ± 0.010 (b)	0.080 ± 0.015 (a)	78.338	0.000
75	32.842	unidentified hydrocarbon	0.097 ± 0.040 (b)	0.202 ± 0.064 (b)	0.506 ± 0.071 (a)	64.247	0.000
76	32.878	unidentified hydrocarbon	None	None	0.014 ± 0.004	28.607	0.000
77	32.915	unidentified hydrocarbon	0.058 ± 0.007 (b)	0.033 ± 0.014 (c)	0.195 ± 0.021 (a)	41.577	0.000
78	32.935	unidentified hydrocarbon	0.065 ± 0.008 (a)	0.076 ± 0.016 (a)	None (b)	41.105	0.000
79	33.030	unidentified hydrocarbon	0.021 ± 0.005 (a)	0.090 ± 0.021 (a)	None (b)	24.595	0.000
80	33.075	C22	0.224 ± 0.008 (b)	0.304 ± 0.023 (a)	0.285 ± 0.014 (a)	34.702	0.000
81	33.158	unidentified	0.024 ± 0.010	0.012 ± 0.006	None	19.317	0.000
82	33.483	unidentified hydrocarbon	0.026 ± 0.006	None	None	20.732	0.000
83	33.604	unidentified hydrocarbon	0.026 ± 0.019 (b)	0.468 ± 0.054 (a)	None (b)	118.439	0.000
84	33.668	unidentified hydrocarbon	0.213 ± 0.039 (a)	None (b)	0.013 ± 0.008 (b)	40.656	0.000
85	33.714	C23	6.121 ± 0.137 (a)	6.499 ± 0.176 (a)	4.133 ± 0.098 (b)	78.208	0.000
86	33.879	11MeC23	0.118 ± 0.005 (b)	0.161 ± 0.014 (a)	0.082 ± 0.005 (c)	41.059	0.000
87	34.015	unidentified hydrocarbon	0.045 ± 0.006 (b)	0.242 ± 0.034 (a)	0.103 ± 0.060 (b)	34.488	0.000
88	34.016	unidentified hydrocarbon	0.006 ± 0.002	0.007 ± 0.007	None	5.356	0.068
89	34.050	9-octadecenamide (78%)	0.177 ± 0.021 (a)	0.206 ± 0.087 (a)	None (b)	38.437	0.000
90	34.245	unidentified hydrocarbon	None	0.184 ± 0.062	None	32.008	0.000
91	34.296	unidentified hydrocarbon	0.015 ± 0.005 (b)	0.166 ± 0.035 (a)	None (b)	48.439	0.000
92	34.306	unidentified hydrocarbon	0.011 ± 0.002 (a)	0.007 ± 0.007 (ab)	None (b)	19.741	0.000
93	34.602	C25:1	1.714 ± 0.063 (a)	1.390 ± 0.069 (b)	1.416 ± 0.086 (b)	18.565	0.000
94	34.633	C25:1	0.176 ± 0.018 (b)	0.386 ± 0.012 (a)	0.023 ± 0.010 (b)	79.611	0.000
95	34.712	C25	5.138 ± 0.140 (a)	5.230 ± 0.192 (a)	4.092 ± 0.168 (b)	35.892	0.000
96	34.869	11MeC25	0.585 ± 0.015 (b)	0.550 ± 0.024 (b)	0.856 ± 0.086 (a)	41.958	0.000
97	34.929	5MeC25	0.048 ± 0.004 (b)	0.030 ± 0.020 (c)	0.115 ± 0.017 (a)	64.733	0.000
98	34.982	2MeC25	0.322 ± 0.070 (b)	0.387 ± 0.051 (a)	0.406 ± 0.017 (a)	62.532	0.000
99	35.026	5,17diMeC25?	0.162 ± 0.009 (b)	0.327 ± 0.025 (a)	0.249 ± 0.011 (a)	56.417	0.000
100	35.050	unidentified hydrocarbon	0.001 ± 0.001	0.063 ± 0.024	None	26.919	0.000
101	35.074	5,15diMeC25	0.250 ± 0.009 (b)	0.209 ± 0.025 (b)	0.325 ± 0.014 (a)	24.967	0.000
102	35.138	C26	0.699 ± 0.073	0.501 ± 0.016	0.554 ± 0.027	0.614	0.735
103	35.197	unidentified hydrocarbon	0.142 ± 0.012 (b)	0.337 ± 0.035 (a)	0.263 ± 0.015 (a)	51.199	0.000
104	35.223	unidentified hydrocarbon	0.020 ± 0.005 (b)	0.171 ± 0.023 (a)	None (b)	71.163	0.000
105	35.258	13MeC26	0.113 ± 0.015 (a)	0.050 ± 0.011 (a)	None (b)	42.924	0.000
106	35.278	unidentified hydrocarbon	0.206 ± 0.014 (a)	0.083 ± 0.015 (b)	0.002 ± 0.002 (c)	82.743	0.000
107	35.300	unidentified hydrocarbon	0.002 ± 0.002 (b)	0.339 ± 0.029 (a)	0.395 ± 0.025 (a)	130.863	0.000
108	35.316	diMeC26?	None	0.010 ± 0.007	None	8.844	0.012
109	35.364	C27:2	0.564 ± 0.035 (b)	0.325 ± 0.035 (c)	0.873 ± 0.047 (a)	57.198	0.000
110	35.395	2MeC26?	0.054 ± 0.019 (b)	0.370 ± 0.104 (a)	None (b)	57.687	0.000
111	35.461	C27:1	0.958 ± 0.036 (a)	0.854 ± 0.030 (a)	0.003 ± 0.003 (b)	95.900	0.000
112	35.490	C27:1	0.222 ± 0.022 (c)	0.405 ± 0.015 (b)	1.154 ± 0.194 (a)	93.526	0.000
113	35.551	C27	12.504 ± 0.513 (b)	16.140 ± 0.724 (a)	8.768 ± 0.389 (c)	53.013	0.000
114	35.592	unidentified hydrocarbon	0.058 ± 0.042	0.205 ± 0.117	0.065 ± 0.065	3.870	0.144
115	35.671	13MeC27+11MeC27+9MeC27	3.730 ± 0.100 (a)	3.496 ± 0.132 (ab)	3.083 ± 0.120 (b)	21.225	0.000
116	35.737	5MeC27?	0.084 ± 0.014 (a)	0.056 ± 0.011 (ab)	0.025 ± 0.011 (b)	15.726	0.000
117	35.770	11,15diMeC27	1.185 ± 0.058 (a)	0.857 ± 0.030 (b)	0.989 ± 0.033 (ab)	11.612	0.003
118	35.821	7,15diMeC27	0.271 ± 0.022 (b)	0.353 ± 0.012 (b)	0.567 ± 0.024 (a)	68.083	0.000
119	35.858	5,17diMeC27+5,13diMeC27?	0.434 ± 0.019 (b)	0.289 ± 0.027 (c)	0.531 ± 0.016 (a)	45.269	0.000
120	35.862	unidentified hydrocarbon	0.003 ± 0.003 (b)	0.182 ± 0.037 (a)	None (b)	67.286	0.000
121	35.921	C28	1.719 ± 0.083 (a)	0.898 ± 0.047 (b)	1.866 ± 0.062 (a)	59.354	0.000
122	35.947	unidentified	None (b)	0.126 ± 0.039 (a)	None (ab)	41.711	0.000

Peak No	Retention Time	Identification	Drones 2-3 days	Drones 7-8 days	Drones 12-15 days	Kruskal-Wallis test	
			mean ± SEM	mean ± SEM	mean ± SEM	H	p
123	35.990	unidentified hydrocarbon	0.043 ± 0.014 (b)	0.175 ± 0.051 (a)	None (b)	25.739	0.000
124	36.038	14MeC28	0.773 ± 0.033 (ab)	0.975 ± 0.108 (a)	0.716 ± 0.016 (b)	11.121	0.003
125	36.156	5,9diMeC28?	0.532 ± 0.021 (b)	0.558 ± 0.042 (ab)	0.618 ± 0.012 (a)	10.156	0.006
126	36.232	C29:1	2.149 ± 0.063	2.227 ± 0.076	2.124 ± 0.041	1.441	0.486
127	36.298	C29	9.685 ± 0.317 (a)	9.595 ± 0.408 (a)	7.457 ± 0.326 (b)	39.910	0.000
128	36.415	13MeC29+11MeC29	4.751 ± 0.107 (a)	4.259 ± 0.172 (b)	4.308 ± 0.139 (b)	13.189	0.001
129	36.527	11,17diMeC29	2.677 ± 0.064 (a)	1.885 ± 0.065 (b)	2.705 ± 0.071 (a)	45.185	0.000
130	36.565	7,19diMeC29?	0.143 ± 0.067 (b)	0.534 ± 0.054 (a)	None (b)	101.600	0.000
131	36.609	5,17diMeC29?	0.812 ± 0.028 (b)	0.592 ± 0.013 (c)	1.077 ± 0.020 (a)	87.021	0.000
132	36.684	C30	1.075 ± 0.053 (b)	0.572 ± 0.020 (c)	1.489 ± 0.031 (a)	98.287	0.000
133	36.728	unidentified hydrocarbon	0.049 ± 0.023 (b)	0.120 ± 0.026 (a)	None (b)	33.760	0.000
134	36.810	14MeC30+13MeC30+11MeC30	0.583 ± 0.029 (b)	0.351 ± 0.012 (c)	0.905 ± 0.085 (a)	78.390	0.000
135	36.862	unidentified hydrocarbon	0.062 ± 0.012 (b)	0.186 ± 0.025 (a)	None (b)	51.566	0.000
136	36.894	unidentified	None	0.091 ± 0.032	None	36.827	0.000
137	36.914	unidentified hydrocarbon	0.020 ± 0.010 (b)	0.104 ± 0.027 (a)	None (b)	30.409	0.000
138	36.895	unidentified	0.029 ± 0.011	None	None	6.641	0.036
139	36.945	C31:2	0.088 ± 0.018 (a)	0.004 ± 0.004 (ab)	None (b)	20.820	0.000
140	36.973	unidentified hydrocarbon	0.621 ± 0.064 (b)	0.460 ± 0.060 (b)	1.460 ± 0.074 (a)	62.652	0.000
141	37.025	C31:1	4.886 ± 0.212 (b)	5.448 ± 0.210 (ab)	6.356 ± 0.341 (a)	21.002	0.000
142	37.049	C31:1	2.818 ± 0.118 (b)	3.771 ± 0.126 (a)	1.976 ± 0.277 (b)	22.494	0.000
143	37.110	C31	4.327 ± 0.105 (b)	4.332 ± 0.142 (ab)	4.726 ± 0.131 (a)	9.814	0.007
144	37.156	unidentified hydrocarbon	0.165 ± 0.048	None	0.076 ± 0.043	5.192	0.074
145	37.243	15MeC31+13MeC31	2.430 ± 0.053 (a)	1.704 ± 0.053 (b)	2.458 ± 0.070 (a)	48.086	0.000
146	37.362	13,17diMeC31	1.478 ± 0.049 (b)	0.916 ± 0.035 (c)	1.737 ± 0.056 (a)	60.423	0.000
147	37.421	unidentified hydrocarbon	0.009 ± 0.007	0.020 ± 0.011	0.124 ± 0.033	15.774	0.000
148	37.480	unidentified hydrocarbon	0.844 ± 0.032 (b)	0.519 ± 0.024 (c)	1.108 ± 0.023 (a)	72.191	0.000
149	37.574	C32	0.583 ± 0.045 (b)	0.111 ± 0.020 (c)	0.939 ± 0.036 (a)	77.466	0.000
150	37.648	unidentified hydrocarbon	0.008 ± 0.004 (b)	0.054 ± 0.014 (b)	0.867 ± 0.060 (a)	126.010	0.000
151	37.737	unidentified hydrocarbon	0.498 ± 0.046	0.359 ± 0.043	0.477 ± 0.060	3.118	0.210
152	37.722	unidentified hydrocarbon	0.094 ± 0.023	None	None	15.024	0.000
153	37.807	unidentified	0.009 ± 0.005	0.003 ± 0.003	None	2.254	0.323
154	37.864	unidentified hydrocarbon	None (b)	0.102 ± 0.025 (b)	0.930 ± 0.058 (a)	122.196	0.000
155	37.914	C33:2	1.400 ± 0.060 (b)	0.857 ± 0.048 (c)	1.848 ± 0.070 (a)	51.229	0.000
156	38.008	C33:1	7.099 ± 0.268 (b)	7.319 ± 0.270 (b)	9.802 ± 0.272 (a)	39.855	0.000
157	38.101	C33	1.601 ± 0.060 (a)	0.759 ± 0.043 (b)	1.601 ± 0.063 (a)	53.186	0.000
158	38.164	unidentified hydrocarbon	0.728 ± 0.071 (a)	0.045 ± 0.026 (b)	None (b)	64.309	0.000
159	38.201	unidentified	0.172 ± 0.060 (b)	0.124 ± 0.042 (b)	0.712 ± 0.153 (a)	36.071	0.000
160	38.268	17MeC31+13MeC31	1.014 ± 0.042 (a)	0.497 ± 0.041 (b)	1.037 ± 0.057 (a)	43.128	0.000
161	38.408	unidentified hydrocarbon	0.273 ± 0.042 (a)	None (b)	0.333 ± 0.033 (a)	28.754	0.000
162	38.466	unidentified hydrocarbon	0.077 ± 0.037 (b)	0.227 ± 0.043 (a)	0.113 ± 0.039 (b)	28.671	0.000
163	38.545	unidentified hydrocarbon	0.106 ± 0.023	None	None	19.560	0.000
164	38.714	unidentified	0.447 ± 0.051 (a)	None (b)	0.346 ± 0.031 (a)	44.351	0.000
165	38.774	unidentified	0.403 ± 0.066 (a)	None (b)	0.078 ± 0.028 (b)	28.008	0.000
166	38.814	unidentified	0.208 ± 0.048 (a)	None (b)	None (b)	26.831	0.000
167	38.900	unidentified	0.041 ± 0.015	None	None	7.641	0.021
168	39.015	unidentified	0.114 ± 0.030	None	0.006 ± 0.006	16.812	0.000
169	39.095	unidentified	None	0.016 ± 0.007	None	22.558	0.000
170	39.130	C35:2	0.244 ± 0.024 (b)	0.025 ± 0.008 (c)	0.450 ± 0.033 (a)	53.297	0.000
171	39.177	unidentified hydrocarbon	None	0.018 ± 0.008	None	22.558	0.000
172	39.240	C35:1	0.364 ± 0.031 (a)	0.120 ± 0.020 (b)	0.456 ± 0.031 (a)	35.260	0.000
173	39.266	unidentified hydrocarbon	0.037 ± 0.012	None	None	9.681	0.007
174	39.342	C35	0.083 ± 0.021	0.071 ± 0.027	0.003 ± 0.003	9.485	0.008
175	39.626	unidentified hydrocarbon	0.086 ± 0.015 (a)	None (b)	0.063 ± 0.014 (a)	17.380	0.000
176	39.835	unidentified	0.047 ± 0.013	None	0.008 ± 0.005	8.139	0.017
177	39.926	unidentified	0.026 ± 0.009	None	None	8.864	0.013
178	40.063	unidentified	0.025 ± 0.009	None	None	9.681	0.007
179	40.224	C36	0.004 ± 0.003 (a)	None (a)	0.070 ± 0.031 (b)	37.103	0.000
180	40.414	unidentified	0.111 ± 0.089	0.026 ± 0.011	None	10.904	0.004
181	40.491	unidentified	0.689 ± 0.199 (a)	0.021 ± 0.007 (ab)	None (b)	32.397	0.000
182	40.600	unidentified	0.297 ± 0.029 (b)	1.659 ± 0.209 (a)	0.134 ± 0.007 (c)	82.513	0.000
183	40.679	unidentified	0.229 ± 0.071	None	0.010 ± 0.008	19.349	0.000
